# Wound Healing Modulation in Glaucoma Filtration Surgery– Conventional Practices and New Perspectives: Antivascular Endothelial Growth Factor and Novel Agents (Part II)

**DOI:** 10.5005/jp-journals-10008-1160

**Published:** 2014-06-12

**Authors:** Jennifer C Fan Gaskin, Dan Q Nguyen, Ghee Soon Ang, Jeremy O'Connor, Jonathan G Crowston

**Affiliations:** Glaucoma Fellow, Glaucoma Investigation and Research Unit, Centre for Eye Research, University of Melbourne, Melbourne, Australia; Consultant, Department of Ophthalmology, Mid Cheshire Hospitals, NHS Foundation Trust, Cheshire; Institute for Science and Technology in Medicine, Keele University, Keele, Staffordshire, UK; Consultant, Glaucoma Investigation and Research Unit, Centre for Eye Research, University of Melbourne, Melbourne, Australia; Consultant, Glaucoma Investigation and Research Unit, University Hospital Limerick, Ireland; Professor, Glaucoma Investigation and Research Unit, Centre for Eye Research, University of Melbourne, Melbourne, Australia

**Keywords:** Glaucoma, Wound healing modulation, Filtration surgery.

## Abstract

Glaucoma filtration surgery is regularly performed for the treatment of glaucoma and trabeculectomy is often regarded as the ‘gold standard' glaucoma operation. The biggest risk of failure of the operation is bleb scarring. The advent of antifibrotic agents, such as mitomycin C (MMC) and 5-fluorouracil (5FU) has vastly prolonged the longevity of the bleb, but concerns remain regarding the potential increase in postoperative complications. More selective therapeutic targets have therefore been explored. One of these is vascular endothelial growth factor (VEGF) inhibition. VEGF inhibition has a role not only in subconjunctival angiogenesis inhibition but also it has direct anti-fibrotic properties. Newer pharmacological compounds and materials have also been developed in recent years in attempt to modulate the wound healing in different ways after glaucoma surgery. These include physical barriers to scarring and vehicles for sustained release of pharmacological agents, and early promising results have been demonstrated. This two-part review will provide a discussion of the application of anti-fibrotic agents in glaucoma filtration surgery and evaluate the newer agents that have been developed.

**How to cite this article:** Fan Gaskin JC, Nguyen DQ, Ang GS, O'Connor J, Crowston JG. Wound Healing Modulation in Glau coma Filtration Surgery–Conventional Practices and New Pers pectives: Antivascular Endothelial Growth Factor and Novel Agents (Part II). J Curr Glaucoma Pract 2014;8(2):46-53.

## INTRODUCTION

Trabeculectomy was introduced by Cairns in 1968 and remains the most commonly performed procedure for the treat ment of glaucoma.^1,2^ The introduction of antifibrotic adjuncts, namely mitomycin C (MMC) and 5-fuoro uracil, has significantly improved the efficacy of the tradi tional fl tering procedure and the indications for and evidence behind the use of antimetabolites were discussed in Part I of this series. However, these substances have also been associated with the potential for increased complication rate of the filtering procedure due to their nonspecific cito-cidal activity, resulting in loss of the conjunctival barrier that can lead to thin-walled avascular blebs with an elevated risk of hypotony and infection. Consequently, alternative, safer forms of wound healing modulation have been sought and, in particular, agents with more specific and targeted activity. Antivascular endothelial growth factor (anti-VEGF) has been identified as a potential solution due to its more selective mode of action. Part II of this series will examine the evidence behind the use of anti-VEGF in trabe culectomy as well as discuss the newer agents/materials being developed.

### The Role of VEGF and Anti-VEGF in Glaucoma Filtration Surgery

Due to the nonspecific inhibitory activity and continued risk of failure associated with antifibrotic agents, other forms of more selective wound modulation with acceptable side effects have been explored in recent years. Vascular endothelial growth factor (VEGF) plays an important role in both physiological and pathological angiogenesis throughout the body. Different VEGF isoforms may be responsible for differential roles in ocular wound healing. Van Bergen et al^3^ assessed the presence of VEGF in trabeculectomy blebs and reported VEGF(165) and VEGF(121) to be predominantly associated with blood vessel growth, and VEGF(189) to be involved in fibrosis. Lopilly Park et al^4^ analyzed the levels of VEGF in both the aqueous humor and Tenon's tissue in patients with primary open angle glaucoma (POAG) and correlated the levels with their outcome following glaucoma surgery. They identified a significant association between the VEGF level in Tenon's tissue at the time of glaucoma surgery with the 1-year intraocular pressure (IOP) and the ultimate success of the operation. Therefore by reducing angiogenesis and subsequent Inflammation and collagen deposition, VEGF inhibition may have a beneficial impact on glaucoma surgical outcome.

Bevacizumab is a recombinant humanized monoclonal immuno globulin antibody of nonselective VEGF. In a cell culture model, bevacizumab demonstrated anti-fibrotic pro perties in a number of ways, including disrup tion of fibro blast proliferation, inhibition of collagen contracti-bility and induction of fibroblast cell death.^5^ Delayed wound healing is also a known complication of systemic bevacizumab, such as the wound dehiscence seen after colo-rectal surgery.^6^ In an early case report, Coote et al^7^ demonstrated significantly reduced bleb vascularity following a single subconjunctival injection of bevacizumab in an eye that had recently undergone cataract surgery 3 months post-MMC trabeculectomy.

### Comparison of the Efficacy of Wound Healing Modulation of Bevacizumab with Conventional Antifibrotic Agents

Unfortunately, the number of studies comparing the efficacy of bevacizumab with conventional antifibrotic agents is small, limited in sample size and often with conficting conclusions.

Niforushan et al^8^ conducted a prospective, randomized study assessing the efficacy of subconjunctival bevacizumab (2.5 mg/0.1 ml; 18 eyes) at the end of trabeculectomy compared with MMC-augmented trabeculectomy (0.002% for 3 minutes; 18 eyes) and demonstrated significantly lower IOP control postoperatively in the MMC group compared to the bevacizumab group (10 mm Hg *vs* 14 mm Hg respectively, p < 0.001). The MMC group also had a higher cumulative probability of total success, although this was not statistically significant.

Sengupta et al^9^ performed a similar pilot study in patients under going combined phacotrabeculectomy by randomizing 38 patients into three groups: (1) conventional single-site phacotrabeculectomy with 0.3 mg/ml intraoperative MMC for 2 to 3 minutes; (2) single-site phacotrabeculectomy with three subconjunctival bevacizumab injections (1.25 mg/ 0.05 ml) performed immediately prior to surgery, immediately following surgery, and on the seventh postoperative day, and (3) single-site phacotrabeculectomy with bevaci zumab-soaked sponge (1.25 mg/0.05 ml) applied intra operatively for 3 minutes to the scleral bed. They reported subconjunctival bevacizumab to be equally effective in reducing intraocular pressure compared with MMC, only with a better safety profile. However, bevacizumab soaked in a sponge appeared to have no advantages over MMC.

When comparing bevacizumab with 5FU, a recent study by Ozgonul et al^10^ analyzed the efficacy of subconjunctival 5FU compared to both subconjunctival and intravitreal bevacizumab in moderating bleb size, vascularity, and IOP postoperatively in an animal model. They observed greater bleb length, width and height in the subconjunctival bevacizumab group compared to the other two groups. Eyes in the subconjunctival bevacizumab group also demons trated significantly lower IOP values compared to the subconjunctival 5FU group, but not compared to the intravitreal bevacizumab group. Inflammation, neovas-cularization and fibrosis were correspondingly less in the subconjunctival bevacizumab group on histological analysis of the blebs.

Jurkowska-Dudzinska et al^11^ reported no significant differences between groups of patients who received four repeated subconjunctival bevacizumab injections (1.25 mg) immediately before and after trabeculectomy and patients who underwent 5FU augmented trabeculectomy without postoperative bevacizumab injections. Simsek et al^12^ com pared subconjunctival bevacizumab (1.25 mg/0.1 ml) and 5FU (5 mg/0.2 ml) in needle revision of failed trabe-culectomy blebs and reported 5FU to be more effective than bevacizumab application.

There is evidence that bevacizumab may act in synergy with 5FU. In a rabbit model of glaucoma fl tra-tion surgery, How et al^13^ reported that combined bevaci-zumab and 5FU offered superior anti-fibrotic effect over mono therapy using either agent, and at the same time prolonging bleb survival. In a randomized controlled trial, Chua et al^14^ obser ved a trend for increased central bleb avas cularity after a single post operative combined injection of bevaci zumab (1.25 mg/0.05 ml) and 5FU (7.5 mg/0.15 ml), compared to 5FU (7.5 mg/0.15 ml) alone. Freiberg et al^15^ compared postoperative subconjunctival 5FU (5 mg/ 0.5 ml) injections with or without one single injection of subconjunctival bevacizumab (3.5 mg/0,14 ml) following MMC-augmented trabeculectomy in 61 patients. They demonst rated a significantly lower nu mber of 5FU injections if bevacizumab was also administered. Whilst there was no difference in mean postoperative IOPs between the two groups, significantly fewer patients in the bevaci zumab group required bleb needling in the follow-up period (mean 25 ± 19 months).

There is currently no conclusive evidence as to the optimal method of delivery of anti-VEGF therapy, with the majority of published studies evaluating either subcon-junctival or intravitreal injection.

### Novel Techniques of Modulating Postoperative Scarring in Glaucoma Filtration Surgery

In recent years, many alternative agents and materials have been investigated for the purpose of wound modulation in glaucoma filtration surgery. In particular, research has focused on novel pharmacological compounds to modulate wound healing, physical barriers to limit scleral-conjunctival fibrosis, and better drug delivery of wound modulating agents. This section will provide a discussion of some of these agents. Most of these agents have only been analyzed through one or two studies and are still in the laboratory phase of development but do demonstrate some promise.

Anti-Placental Growth Factor (Anti-PIGF)

Although anti-VEGF has been demonstrated to improve surgical outcomes, the improvement is thought to occur mostly as a result of inhibition of angiogenesis rather than a ny significant anti-Inflammatory response. ^16,17^ Researchers have hypothesised that upregulation of placental growth factor may account for the persistent Inflammatory response postfiltration surgery that cannot be inhibited by either selective or nonselective anti-VEGF.^18-20^ Placental growth factor (PIGF) is a VEGF-homologue that binds solely to VEGF-R1.^21^ It is notinvolve dinphy siological angiogenesis and acts only on pat hological angiogenic and in fammatory processes.^22,23^ In a multifaceted study conducted by Van Bergen et al,^24^ the authors demonstrated that PIGF was upregulated by 40% in the aqueous humor of human eyes with glaucoma compared to nonglaucomatous eyes; this upregulation was locally produced as the increased PIGF was not demonstrated in the serum of the same patients. The authors also reported a further enhanced upregulation of PIGF if intravitreal bevacizumab was administered. In a mouse model of glaucoma filtration surgery, the authors reported improved surgical outcome with increasing bleb survival and bleb area following intracameral injection of anti-PIGF. This was associated with a significant reduction in cellular proliferation, Inflammation and angiogenesis during the first postoperative days after surgery, and with a decrease in collagen deposition at the later stages. They hypothesized that PIGF may be a potential therapeutic target for glaucoma surgery, and proposed that a combination of optimal-dosing anti-PIGF with suboptimal-dosing anti-VEGF may be more effective at reducing scar formation compared to monotherapy with either agent alone.

Infiximab

Tumor ne crosis factor alpha (TNF- α) ha s also been identi fe d as a potential target for wound healing modulation in glaucoma filtration surgery. It is a local paracrine and autocrine regulator for low-density leukocyte and endothelial cells. It stimulates mononuclear phagocytes as well as other cell types that produce various proInflammatory cytokines and chemokines, and induces migration of polymorphic nuclear leukocytes. Infiximab is a mouse/human chimeric monoclonal IgG1 antibody against TNF-α. It binds to TNF-α and reduces both lymphocyte migration and production of proInflammatory cytokines. Its use in systemic inflammatory disorders is well-documented,^25^ and it is increasingly employed to treat ocular inflammatory conditions.^26,27^ Turgut et al^28^ investigated the efficacy of post-trabeculectomy topical infiximab eye drops (10 mg/ml, four times a day for 14 days) compared with (1) intraoperative MMC 0.4 mg/ml for 3 minutes, (2) trabeculectomy with postoperative saline drops (four times a day for 14 days) (sham group), and (3) no treatment (control group) in 28 rabbits. At day 14, the rewere higher mean fibroblast and mononuclear cell num bers as well as higher intensities of immunofuorescent staining with transforming growth factor-β (TGF-β), fibroblast growth factor-β (FGF-β), and platelet-derived growth factor (PDGF) in the sham group compared to the control group. The mean Inflammatory cell counts and immunostaining intensities TGF-β, FGF-β, a nd PDGF were lower in bot h the MMC group and infiximab group compared to the sham group, but there was no significant difference between the MMC group and infiximab group.

Trastuzumab

The same investigators also assessed the effects of subcon-junctivally-administered trastuzumab in the rabbit model of filtration surgery.^29^ Trastuzumab is a humanized monoclonal antibody against the extracellular domain of the human epidermal growth factor 2 (HER2). It is most widely used in the treatment of overexpression of HER2 in early or metastatic breast cancer, although its mechanism of action is not completely understood.^30^ When compared to trabe-culectomy combined with intrascleral application of MMC (0.4 mg/ml for 3 minutes), trabeculectomy with a subcon-junctival injection of trastuzumab (1.2 mg/0.1 ml) showed comparable numbers of mononuclear cells and fibroblasts in the filtration site on histology (p > 0.05). Similarly, the immunostaining intensities of TGF-β, FGF-β, and PDGF were also comparable between the two groups (p > 0.05).

Connexin43 Antisense Oligodeoxynucleotides (Cxn43 AsODN)

Gap junctions are structures that allow direct signalling between cells.^31^ They play a role in in fammation, ^32^ cell migra-tion^33^ and tissue contraction.^34^ Gap junctions have a complex structure: Six connexin protein subunits oligomerize to form a hemichannel called a connexon; two connexons from neighboring cells dock to form a complete intercellular junction channel. Multiple intercellular channels cluster together to form gap junctions with the number of channels in a plaque varying considerably.^31^ Connexin43 (Cxn43) is one of the most ubiquitous and studied isoforms.^35,36^ Connexin43 antisense oligodeoxynucleotides (Cxn43 AsODN) has been demonstrated to cause a transient reduc tion in Cxn43 protein expression and improve cutaneous wound healing. Coutinho et al^37^ reported faster wound closure and smaller scar formation following partial-thickness burn wounds in mice after application of Cxn43 AsODN. Qiu et al^38^ also demonstrated reduced Inflammation, both macroscopically and microscopically, in addition to improved wound healing following one application of Cxn43 AsODN in both incisional and excisional cutaneous wounds in rats. Deva et al^39^ compared the histological outcome of trabeculectomy combined with a subconjunctival injection of Cxn43 AsODN in pluronic gel with an injection balanced salt solution or pluronic gel into the formed bleb of rabbit eyes. They observed that eyes that received Cxn43 AsODN had reduced Cx43 upregulation at 8 and 24 hours post-injection, which led to less myofibroblast upregulation at days 5 and 21 and reduced scarring at day 21 compared to controls.

Physical Barriers

The concept of using amniotic membrane during glaucoma filtration surgery was first described in 1998.^40^ In addition to acting as a physical barrier, amniotic membrane also has additional anti-Inflammatory and anti-fibrotic properties, which may further reduce the risk of scar formation. Trabe cu-lectomy combined with amniotic membrane trans plantation and MMC has shown very promising outcomes in terms of IOP control and complications for eyes with refractory glaucoma^41,42^ and eyes considered at high risk of surgical failure.^43^ However, other studies, including two random ized clinical trials, assessing primary trabeculectomy with amniotic membrane *vs* primary trabeculectomy alone (without the use of MMC or 5FU) for POAG have demons-t rated no sign ifica nt benefit in IOP cont rol at the 1 and 2-year time points.^44-46^ These differences in reported out comes may partly be due to the different techniques of amniotic membrane placement as well as the use of antimeta bolites. Nevertheless, the evidence base for the use of amniotic membrane in glaucoma filtration surgery currently remains inconclusive.

Expanded polytetrafuoroethylene (Gore-Tex) membrane has been assessed in glaucoma filtration surgery with some promising results.^47-49^ It is a nonabsorbable material that reduces tissue adhesion and fibrous cap sule thick ness. However, a ra ndomized clinical trial found no significant benefit in IOP control or complication rates in primary trabeculectomy with or without expanded polytetra fuoroethylene membrane placed underneath the scleral fap.^50^

Various other flms and materials have also been evaluated for their ability to form a physical barrier and pre vent adhesions between the sclera and the conjunctiva, mostly in rabbit models. Sepraflm is a biodegradable solid flm composed of hyaluronic acid and carboxymethyl cellulose. Studies of trabeculectomy have shown Sepraflm to reduce adhesions around the conjunctiva and sclera resulting in more prominent bleb formation and lower mean IOP compared to without Seprafilm.^51,52^ When compared to Interceed, an oxidated regenerated cellulose matrix, Sepraflm demonstrated no significant difference in IOP or bleb appearance but unfortunately, neither seemed to be able to significantly suppress the wound healing reaction following trabeculectomy.^53^ Correspondingly, when Interceed was evaluated in trabeculectomy alongside Surgical, another oxidated regenerated cellulose matrix, both had similar outcomes in terms of IOP, bleb appearance and lack of effect on fibroblast proliferation.^54^ However, a more promising option may be trabeculectomy with a honeycomb-patterned biodegradable film composed of poly(L-lactide-co-ε-caprolactone). This has been demonstrated to produce comparable IOP reduction and bleb survival to MMC-en hance d t r abeculectomy, but wit hout the associated complications.^55^

OloGen is a bioengineered, biodegradable, porous collagen-glycosaminoglycans matrix implant that is placed on top of the scleral fap and under Tenon's capsule during trabeculectomy. It was developed as a means of providing controlled resistance between the anterior chamber and the subconjunctival space in the early postoperative period. In addition, its porous structure encourages conjunctival fibroblasts and myofibroblasts to grow into the pores and secrete connective tissue in the form of a loose matrix. As a result, it reduces scar formation and wound contraction and eliminates the need for adjunctive antimetabolites.^56,57^ OloGen degrades naturally within 90 to 180 days of implantation.

In the rabbit model,^56^ significantly lower IOP was seen up to 28 days in eyes that underwent trabeculectomy with OloGen implant compared to unaugmented trabeculectomy. Histological analysis of the surgical site demonstrated loose, randomized collagen fibers in the conjunctival bleb and an unhealed scleral tunnel in the OloGen group compared to dense collagen deposition in the subconjunctival space in the control group. In a pilot study,^58^ 40 eyes of 30 patients were randomized to either trabeculectomy with OloGen implant or unaugmented trabeculectomy alone and followed for 6 months. There was no statistically significant difference in IOP control between the two groups at 6 months: the mean IOP was comparable between the two groups (15.3 ± 4.1 and 15.3 ± 3.7 mm Hg, trabeculectomy with OloGen and unaugmented trabeculectomy respectively), and 90% from each group achieved complete success of achieving IOP ≤ 21 mm Hg without medication. Although not statistically significant, one eye from the OloGen group developed endophthalmitis 10 days after initial surgery and required vitreoret inalinte rvention . Five eyes from the control group and two eyes from the OloGen group developed bleb encystment at 1 month post-trabeculectomy and all patients required treatment with subconjunctival 5FU injections postoperatively (number of injections ranged 2-7).

Subsequently, several randomized controlled trials have compared MMC-augmented trabeculectomy with OloGen-implanted trabeculectomy with varying results. In the largest RCT evaluating 40 eyes of 40 patients, Cillion et al found no significant difference in terms of IOP control and complications between the two groups at up to 24 months' follow-up.^57^ In 39 eyes of 33 subjects, Senthill et al^59^ observed lower IOP in the MMC group at 6 months compared to the OloGen group, but no significant difference at 12 or 24 months. On the contrary, Rosentreter et al^60^ demonstrated significantly lower IOP in MMC-augmented trabeculectomy compared to OloGen-augmented trabe culectomy in 20 eyes of 20 patients at 1 year; 100% of eyes in the MMC group achieved complete success of IOP ≤18 mm Hg and only 50% of eyes in OloGen groups achieved complete success at 1 year. However, they also reported more avascularity in the MMC-augmented blebs than the OloGen-treated blebs in this study.^60^

The use of OloGen implantation has also extended to phacotrabeculectomies and Ex-Press shunts, again with varying reported results when compared to MMC.^61,62^

Another biodegradable implant composed of collagen glycosaminoglycan matrices (CGM) has been introduced for the treatment of post-trabeculectomy hypotony. The implant is inserted subconjunct ivally t h roug h an incision adjacent to the bleb and placed over the scleral fap. In a pilot study of 12 eyes, IOP increased from a mean of 4 to 10 mm Hg after a naver age follow-up of 6 months. ^63^ In this study, one patient developed implant exposure, one developed a corneal dellen adjacent to the implant, and two patients required topical hy poten sive medication at the end of t he follow-up per iod.^63^

Drug Delivery of Current Pharmacological Agents

Alternative methods of delivering antimetabolites in a sus tained yet safe manner was discussed in Part I of this review. In this section, the focus will be on novel forms of drug delivery of other pharmacological agents.

As previously discussed in Part I, various hydrogels have been evaluated in animal models for their ability to provide slow, sustained release of incorporated wound healing modulators. In addition to anti-fibrotic agents, hydrogels have also been developed to incorporate other phar macological agents for use in animal trabeculectomy models. Carbopol 980 hydrogel loaded with paclitaxel demonstrated anti-fibrotic effects on rabbit conjunctival wound healing that was similar to that of MMC alone.^64^ In canine trabeculectomy, implantation of a gelatin hydrogel chymase inhibitor combination reduced cell proliferation and better maintained IOP at 12 weeks compared to controls.^65^ Other novel hydrogels include a bioactive self-assembled peptide that can be used either alone or loaded with 5FU in glaucoma filtration surgery.^66,67^ These hydrogel-compounds are still in the experimental phase.

A new bevacizumab-loaded polyurethane subconjunctival implant has been developed in a rabbit model designed to be inserted adjacent to the filtration site on the episclera.^68^ Compared to the rabbit eyes with non-bevacizumab-loaded polyurethane implants, the eyes with bevacizumab-loaded implants did not demonstrate significant differences in bleb area *in vivo* or collagen density *ex vivo*. However, there was a significantly lower number of VEGF-expressing fibroblasts in the bevacizumab group.

The interest in sodium hyaluronate stems from its utility in sustained drug delivery as well as its in herent anti fibrotic properties and ability to act as a physical spacer. When loaded with dexamethasone, it allowed extended release of the steroid at concentrations sufficient for antiproliferative activity over a period of nearly 2 days.^69^ Two randomized clinical trials evaluated the benefit of subconjunctival hyaluronate in glaucoma surgery: the first at the conclusion of trabeculectomy,^70^ and the second when performed in conjunction with bleb needling for failing trabeculectomy blebs.^71^ Lopes et al reported no significant difference in success rates when comparing subconjunctival sodium hyaluronate 2.3% against balanced salt solution at the conclusion of trabeculectomy after a mean follow-up of 12 months.^70^ Naravanaswamy et al compared a 5FU and hyaluronate 2.3% mixture *vs* 5FU alone in bleb needling of failing blebs and found no significant differences in outcomes at 3 months although the 5FU alone group required repeat needling more frequently.^71^ Aretrospective case series with longer follow-up of 12 months found of bleb needling with subconjunctival sodium hyaluronate 1.4% and 5FU to be effective for reducing intraocular pressure in the medium term with few serious complications.^72^

Another approach that has received keen research interest is microflms loaded with pharma cological agents. Microflms composed of poly (D-, L-lactide-co-capro lactone) and loaded with pred nisolone acetate provi ded steady, sust ained release of prednisolone *in vitro*; subconjunctival implantation reduced postoperative Inflammation and prolonged bleb survival in rabbit trabeculectomy.^73^ A sirolimus sustained-delivery flm composed of polyactioglyconic acid polymer also showed similar anti-Inflammatory effects after filtration surgery in a rabbit model.^74^

## CONCLUSION

Glaucoma filtration surgery has an indisputable place in the management of glaucoma but efforts to minimize com-pli cations associated with antifibrotic use remain ongoing. Significant research is being conducted on newer, alternative forms of trabeculectomy wound modu lation. Agents such as anti-VEGF and other novel pharmacological compounds have shown promise, but require confirmation and refinement in application through larger, prospective clinical trials. It is an exciting evolving era for glaucoma wound modulation, and undoubtedly the humble trabeculectomy will be undergoing a facelift in the near future.
